# Exercise and Epigenetic Regulation in COPD: Current Evidence and Potential Mechanistic Pathways

**DOI:** 10.3390/ijms262311392

**Published:** 2025-11-25

**Authors:** Yuanming Zhong, Jianhua Xu, Xia Chen, Yi Lv, Xi Zheng

**Affiliations:** 1 School of Physical Education and Sport Sciences, Fujian Normal University, Fuzhou 350117, China; 13007958372@163.com (Y.Z.); xujianhua@fjnu.edu.cn (J.X.); 13559187397@163.com (X.C.); 2 Fujian Key Laboratory of Developmental and Neural Biology, College of Life Sciences, Fujian Normal University, Fuzhou 350117, China

**Keywords:** chronic obstructive pulmonary disease, exercise, pulmonary rehabilitation, epigenetics, DNA methylation, histone modifications, non-coding RNAs

## Abstract

Chronic obstructive pulmonary disease (COPD) remains a major global health burden, characterized by persistent airflow limitation, progressive lung tissue destruction, and systemic comorbidities. Conventional pharmacological therapies alleviate symptoms but have limited efficacy in halting disease progression. Epigenetic mechanisms, including DNA methylation, histone modifications, and non-coding RNAs, are increasingly recognized as important regulators of the inflammatory and oxidative pathways involved in COPD pathogenesis. Exercise is a cornerstone of pulmonary rehabilitation and consistently improves exercise capacity, symptoms, and systemic inflammatory markers in COPD. Emerging studies suggest that exercise is associated with changes in epigenetic regulators. This review summarizes current clinical and preclinical findings on how aerobic training, resistance exercise, and high-intensity interval training are linked to alterations in DNA methylation, histone marks, and non-coding RNA expression, and discusses the biological plausibility that these changes could influence COPD-related pathways such as antioxidant responses, protease and antiprotease balance, and inflammatory signaling. Critical gaps remain regarding tissue specificity, temporal dynamics, and causal mechanisms. Future research should prioritize longitudinal and mechanistic studies to clarify whether epigenetic responses contribute to the benefits of exercise in COPD and to assess their potential as biomarkers or therapeutic targets.

## 1. Introduction

Chronic obstructive pulmonary disease (COPD) is a leading cause of morbidity and mortality worldwide, affecting more than 300 million individuals and contributing to over 3 million deaths annually [1]. It is characterized by persistent airflow limitation, chronic airway inflammation, parenchymal destruction, and progressive decline in pulmonary function [2,3]. Despite advances in pharmacological therapy, including bronchodilators, corticosteroids, and emerging biologics, no available treatment can reverse structural lung damage or halt disease progression. In addition, COPD is frequently accompanied by systemic manifestations such as skeletal muscle dysfunction, cardiovascular complications, and metabolic disorders, which further exacerbate its disease burden and reduce quality of life [4,5,6,7]. These challenges underscore the urgent need to explore novel therapeutic approaches that extend beyond symptomatic management.

Recent years have witnessed growing interest in the role of epigenetics in the pathogenesis of COPD. Epigenetic mechanisms including DNA methylation, histone modifications, and non-coding RNAs (ncRNAs) act as critical regulators of gene expression without altering the DNA sequence. Environmental exposures such as cigarette smoke (CS), air pollution, and oxidative stress can induce persistent epigenetic alterations, leading to dysregulated inflammatory and immune pathways. For instance, aberrant DNA methylation has been linked to impaired antioxidant defenses and enhanced pro-inflammatory signaling [8]; histone modifications contribute to chromatin remodeling that sustains inflammatory gene transcription [9,10]; and dysregulated ncRNAs further modulate processes such as apoptosis, extracellular matrix degradation, and immune cell activation [11,12,13]. Collectively, these epigenetic changes establish a molecular memory that drives chronic inflammation and irreversible tissue injury in COPD.

Parallel to these mechanistic insights, physical exercise has long been recognized as a cornerstone of non-pharmacological COPD management. Pulmonary rehabilitation programs incorporating aerobic exercise, resistance training, and breathing exercises improve exercise capacity, reduce dyspnea, and enhance quality of life. Beyond functional outcomes, emerging evidence suggests that exercise exerts systemic anti-inflammatory and antioxidant effects, modulates metabolic homeostasis, and attenuates skeletal muscle wasting [14,15,16,17]. However, the molecular basis of these protective effects remains incompletely understood. Increasingly, studies point to epigenetic modulation as a key mechanism through which exercise exerts its beneficial impact in chronic diseases [18,19,20,21]. Exercise has been shown to reprogram DNA methylation patterns, remodel histone acetylation, and regulate ncRNAs across various tissues, thereby influencing inflammation, oxidative stress, and tissue remodeling—all central processes in COPD pathogenesis.

Despite these promising observations, the epigenetic underpinnings of exercise-mediated protection in COPD remain underexplored. Few studies have systematically examined how different exercise modalities such as aerobic training, resistance training, or high-intensity interval training (HIIT) differentially influence epigenetic regulators in the context of COPD. Moreover, there is a paucity of evidence linking specific epigenetic biomarkers to clinical improvements observed in patients undergoing exercise-based interventions. Addressing these gaps is crucial for advancing precision medicine, as integrating epigenetic signatures into rehabilitation strategies could help stratify patients, monitor treatment response, and identify novel therapeutic targets.

In this review, we synthesize current evidence on the role of epigenetic mechanisms in COPD pathogenesis and highlight how exercise may exert protective effects through epigenetic regulation. We first outline the molecular mechanisms of epigenetic dysregulation in COPD, followed by a discussion of the clinical benefits of different exercise modalities. We then explore emerging evidence for exercise-induced modulation of DNA methylation, histone modifications, and ncRNAs in COPD. Finally, we discuss the translational implications of these findings and propose future directions for research aimed at harnessing epigenetic insights to optimize exercise-based interventions in COPD.

## 2. Epigenetic Landscape of COPD Pathogenesis

**Figure 1:**
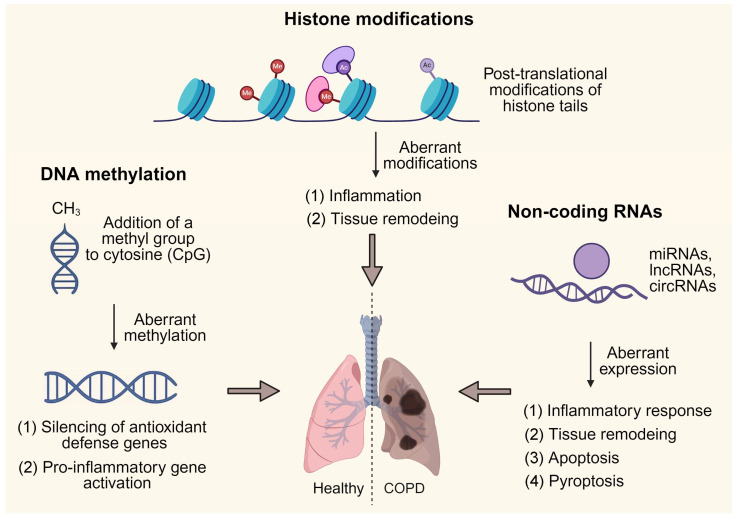
Epigenetic regulation in the pathogenesis of COPD. Created with BioRender.com, der, b. (2025) https://BioRender.com/oikyxt2 (accessed on 4 November 2025).

Epigenetic regulation has emerged as a pivotal mechanism linking environmental exposures to the persistent pathological features of COPD. Unlike genetic mutations, which are irreversible, epigenetic changes are dynamic and can be modified by external stimuli such as CS, pollutants, diet, and physical activity. These changes affect chromatin structure and gene transcription without altering the DNA sequence, thereby establishing a long-lasting molecular “memory” that sustains chronic inflammation, oxidative stress, and tissue remodeling. In COPD, aberrant epigenetic patterns have been observed across multiple molecular layers, including DNA methylation, histone modifications, and ncRNAs. Each of these mechanisms contributes uniquely to the initiation and progression of airway inflammation, alveolar destruction, and impaired repair processes (Figure 1). Understanding the interplay of these epigenetic regulators provides critical insights into COPD pathogenesis and may uncover novel therapeutic targets, particularly in the context of non-pharmacological interventions such as exercise.

### 2.1. DNA Methylation in COPD Pathogenesis

DNA methylation, the addition of a methyl group to cytosine residues within CpG dinucleotides, is one of the most extensively studied epigenetic modifications in COPD. It serves as a key regulator of gene expression by silencing promoters or altering enhancer activity, thereby shaping transcriptional programs relevant to inflammation and tissue homeostasis [22]. Aberrant DNA methylation patterns have been consistently observed in lung tissue, airway epithelial cells, and peripheral blood of COPD patients. For example, hypermethylation of antioxidant defense genes, such as *NFE2L2* and *HOXA5*, leads to impaired cellular responses against oxidative stress [8,23], while hypomethylation of pro-inflammatory genes such as *IL-8* and *TNF-α* promotes sustained neutrophilic inflammation [24]. CS, the most critical risk factor for COPD, induces widespread methylation alterations that persist long after smoking cessation, contributing to the “epigenetic memory” of lung injury [25,26,27,28,29,30,31,32].

Genome-wide methylation profiling has revealed extensive abnormalities across lung compartments, implicating diverse genes and pathways in COPD [33]. For example, differentially methylated regions have been identified in *CHRM1*, linked to PI3K–Akt signaling and airway constriction, as well as in *GLT1D1* and *DTX1*, associated with leukotriene responses and Notch signaling [34]. In small airways, hypermethylation and downregulation of stress-response genes such as *PTEN*, *Nrf2/ARE*, and *IL17F* have been reported [8]. Importantly, these alterations are highly cell type–specific, with airway fibroblasts exhibiting substantially more differentially methylated regions than parenchymal fibroblasts, highlighting heterogeneity in epigenetic regulation across lung tissues [35].

Building on these findings, epigenome-wide association studies (EWAS) have identified COPD-specific methylation signatures that correlate with disease severity, lung function decline, and exacerbation risk [36,37,38]. Collectively, these findings underscore DNA methylation as a central mechanism in COPD pathogenesis, mediating both global and cell type–specific effects, while providing molecular substrates for the long-term impact of environmental insults and the heterogeneity of disease progression.

### 2.2. Histone Modifications in COPD Pathogenesis

Histone modifications represent another critical layer of epigenetic regulation that directly influence chromatin accessibility and transcriptional dynamics. Post-translational modifications, including acetylation, methylation, phosphorylation, and ubiquitination of histone tails, alter nucleosome architecture and determine whether specific genomic regions are transcriptionally active or repressed [39]. In COPD, dysregulation of histone acetylation has been extensively studied [40,41]. CS–induced oxidative stress reduces the activity of histone deacetylases (HDACs), particularly HDAC2, leading to hyperacetylation of pro-inflammatory gene promoters and sustained transcription of cytokines such as IL-8 and CXCL1 [42,43,44,45]. This mechanism not only drives chronic airway inflammation but also contributes to corticosteroid resistance observed in severe COPD patients.

Histone methylation further shapes COPD pathology. Altered levels of histone methyltransferases and demethylases modulate repressive (e.g., H3K27me3) and activating (e.g., H3K4me3) marks, thereby influencing genes related to extracellular matrix degradation, cellular senescence, and immune regulation [43]. Beyond histone tail modifications, ATP-dependent chromatin remodeling complexes (e.g., SWI/SNF family) have also been implicated in COPD, as they reposition nucleosomes to regulate accessibility of stress-responsive genes [46]. Together, these mechanisms converge to establish a pro-inflammatory and proteolytic milieu in the lung, amplifying tissue injury and repair failure. Importantly, because histone modifications are reversible, they represent promising therapeutic targets, with ongoing investigations into HDAC activators, bromodomain inhibitors, and other epigenetic drugs as potential treatments for COPD.

### 2.3. Non-Coding RNAs as Epigenetic Regulators in COPD Pathogenesis

NcRNAs, encompassing microRNAs (miRNAs), long non-coding RNAs (lncRNAs), and circular RNAs (circRNAs), have emerged as pivotal epigenetic regulators in COPD. These molecules fine-tune gene expression post-transcriptionally and participate in multiple signaling cascades that drive inflammation, oxidative stress, apoptosis, and tissue remodeling in the lung.

MicroRNAs are the most extensively studied class in COPD. Aberrant expression of miRNAs, such as the upregulation of miR-15b, miR-21, miR-133, miR-181a, miR-206, miR-223, miR-499, and miR-638, and the downregulation of miR-1, miR-218 and miR-146a, has been linked to exaggerated inflammatory responses and defective immune regulation [47,48,49,50,51,52,53,54,55]. For instance, miR-21 contributes to COPD pathogenesis by modulating apoptosis and inflammation through the PTEN/Akt/NF-κB pathway [56], while loss of miR-146a plays a pathogenetic role in the abnormal inflammatory response in COPD [50].

LncRNAs further expand the complexity of COPD epigenetics by acting as scaffolds, sponges, or guides for chromatin-modifying complexes [57]. A growing number of lncRNAs has been found in the plasma, serum, sputum, bronchial lavage fluid, and lung tissue of COPD patients, which contribute to disease progression through diverse mechanisms [58,59,60,61]. For example, LASI lncRNA is upregulated in the airways of smokers and CSE-exposed macaques, contributing to mucus hypersecretion and goblet cell proliferation [62]. lnc-IL7R downregulation is linked to airway epithelial inflammation and impaired lung function [63]. LINC00612 and HOXA-AS2 are decreased in COPD lungs and regulate endothelial proliferation via Notch1 signaling, which is essential for lung homeostasis, damage, and repair [64,65]. LncRNA Nqo1-AS1 alleviates CS–induced oxidative stress by stabilizing Nqo1 mRNA [66], while lncRNA GAS5 promotes inflammation through the miR-223-3p/NLRP3 axis [60]. LncRNA MIAT enhances airway remodeling via the miR-29c-3p/HIF3A pathway [67], lncRNA OIP5-AS1 silencing mitigates CSE-induced apoptosis and inflammation through the miR-410-3p/IL-13 axis [68], and lncRNA CASC2 overexpression protects against epithelial cell injury by targeting the miR-18a-5p/IGF1 axis [69]. These findings indicate that CS–induced dysregulation of lncRNAs disrupts the balance between inflammation, oxidative stress, and structural cell replenishment, thereby driving COPD pathogenesis. However, functional in vivo evidence remains limited and warrants further investigation.

CircRNAs, though more recently studied, also contribute to COPD pathogenesis. By acting as miRNA sponges, circRNAs regulate the availability of miRNAs and thereby control inflammatory gene networks [70,71]. Recent profiling studies revealed thousands of circRNAs differentially expressed in the lung tissue, plasma, and PBMCs of COPD patients compared to controls [72,73,74,75,76]. Functional analyses indicated that dysregulated circRNAs are involved in key pathogenic processes, including inflammation, oxidative stress, apoptosis, and pyroptosis, partly through pathways such as MAPK, NOD-like receptor, Toll-like receptor signaling, and Th1/Th2 differentiation [72,73,77,78]. Specific circRNAs, such as circ_0008833 (promoting pyroptosis via Caspase-1/IL-1β/IL-18 signaling), circ_0006892 (protective via miR-24/PHLPP2 regulation), and circ_ANKRDII (pro-inflammatory through miR-145-5p inhibition), exemplify their diverse roles in COPD development [73,77,78]. Moreover, due to their structural stability, circRNAs such as circ_0049875, circ_0042590, and others have emerged as promising circulating biomarkers for COPD diagnosis and prognosis [75,76]. These findings suggest that circRNAs not only participate in COPD pathogenesis but may also serve as potential biomarkers and therapeutic targets.

Collectively, ncRNAs function as dynamic regulators linking environmental exposures, such as CS, to epigenetic alterations in gene expression. Their stability in body fluids makes them attractive biomarkers for COPD diagnosis and progression, while therapeutic modulation of ncRNAs holds promise for restoring epigenetic balance in diseased lungs.

## 3. Exercise in COPD: Benefits and Mechanisms

Exercise training is a cornerstone of pulmonary rehabilitation and represents one of the most effective non-pharmacological interventions for COPD. Unlike pharmacotherapy, which mainly provides symptomatic relief and targets acute exacerbations, exercise addresses the systemic manifestations of COPD, including skeletal muscle dysfunction, reduced exercise capacity, and impaired quality of life. A growing body of evidence indicates that structured physical activity, whether aerobic, resistance-based, or interval training, not only improves functional outcomes but also exerts profound effects on molecular pathways implicated in COPD progression.

At the physiological level, exercise enhances ventilatory efficiency, increases oxygen utilization, and improves skeletal muscle endurance, thereby reducing dyspnea and fatigue [79,80,81,82]. Importantly, exercise also modulates systemic inflammation, oxidative stress, and mitochondrial dysfunction—pathological hallmarks closely intertwined with COPD severity [15,82,83,84]. Recent studies have expanded this perspective by linking exercise to epigenetic remodeling, suggesting that physical activity may restore homeostasis in COPD partly through regulation of DNA methylation, histone modifications, and ncRNA networks.

**Figure 2:**
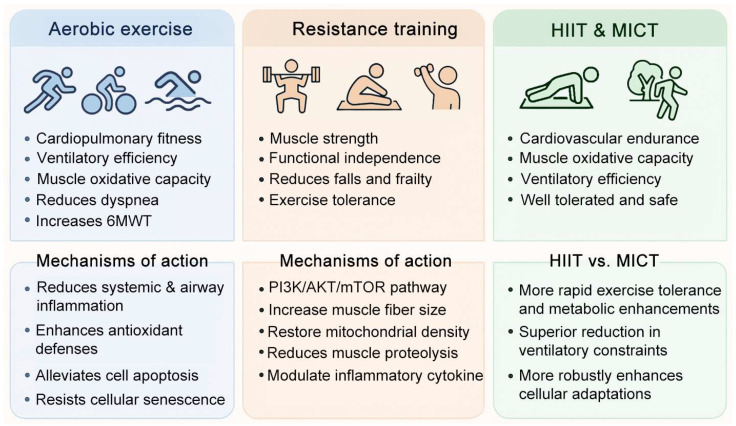
Exercise modalities in COPD rehabilitation: outcomes and mechanisms.

Given the heterogeneity of COPD and variability in patient response, different exercise modalities may confer distinct benefits. Aerobic exercise has been shown to improve cardiovascular fitness and anti-inflammatory capacity, resistance training to counteract muscle atrophy and weakness, and HIIT to induce rapid metabolic and respiratory adaptations. Thus, exploring the differential and overlapping effects of these exercise forms is essential for optimizing rehabilitation strategies. This section will summarize the current evidence on exercise-mediated improvements in COPD, setting the stage for mechanistic discussions on how epigenetic regulation contributes to these protective effects (Figure 2).

### 3.1. Aerobic Exercise–Mediated Mechanisms in COPD

Aerobic exercise, such as walking, cycling, and treadmill training, constitutes the most extensively studied form of physical activity in COPD rehabilitation. Its primary benefits are linked to improvements in cardiopulmonary fitness, ventilatory efficiency, and muscle oxidative capacity, all of which directly alleviate exertional dyspnea and enhance patients’ quality of life. Clinical trials consistently demonstrate that moderate-to-high intensity aerobic training reduces symptoms, increases six-minute walk distance, and lowers hospitalization risk in COPD patients [82,85,86,87,88,89].

Beyond functional outcomes, aerobic exercise exerts systemic effects on the underlying pathophysiology of COPD. Regular training attenuates systemic and airway inflammation, as evidenced by reductions in circulating C-reactive protein, TNF-α, and IL-6 levels [88,89,90,91]. In experimental models, aerobic exercise enhances antioxidant defenses by upregulating superoxide dismutase and catalase, thereby mitigating oxidative stress induced by CS exposure [92,93].

Evidence from animal studies provides mechanistic insight into these protective effects. In CS–exposed mouse models of COPD, treadmill running has been shown to alleviate the inflammatory response, oxidative stress injury, and cell apoptosis [94,95]. Moreover, preclinical data indicate that aerobic training preserves telomere length and reduces markers of cellular senescence in lung tissues, suggesting its role in counteracting premature aging—a hallmark of COPD pathology [96,97,98]. Importantly, animal models reveal that exercise can reprogram gene expression profiles associated with oxidative stress, inflammation, and apoptosis, reinforcing the concept that its benefits extend beyond symptom relief to disease modification [99].

Emerging evidence also highlights the potential role of aerobic exercise in modulating corticosteroid sensitivity. By restoring HDAC2 activity and reducing NF-κB–driven transcription of pro-inflammatory mediators, aerobic training may partially overcome the steroid resistance frequently observed in COPD [100]. Collectively, these findings suggest that aerobic exercise provides multifaceted protection in COPD, not only by improving pulmonary and systemic physiology but also by intervening in key molecular pathways that drive disease progression.

### 3.2. Mechanistic Role of Resistance Training in COPD

Resistance training, encompassing weightlifting, elastic band exercises, and bodyweight training, has emerged as a vital complement to aerobic exercise in COPD rehabilitation. Unlike aerobic training, which primarily targets cardiovascular endurance, resistance exercise directly addresses peripheral muscle weakness and atrophy—systemic manifestations strongly associated with disease severity and poor prognosis in COPD patients. Clinical studies have shown that resistance training significantly improves muscle strength, enhances functional independence, and reduces the risk of falls and frailty in patients with moderate-to-severe COPD [101,102,103,104,105,106,107,108].

At the physiological level, resistance training stimulates muscle hypertrophy by activating the PI3K/Akt/mTOR signaling pathway, thereby increasing protein synthesis and counteracting catabolic processes driven by chronic inflammation [109,110,111,112]. Patients undergoing structured resistance programs demonstrate improved quadriceps cross-sectional area and enhanced maximal voluntary contraction, both of which contribute to greater exercise tolerance [113]. Animal models further confirm the protective role of resistance-type training. Ladder-climbing or weighted-squat training protocols increase muscle fiber size, restore mitochondrial density, and reduce markers of muscle proteolysis such as atrogin-1 and MuRF1 [114,115,116,117]. Beyond musculoskeletal adaptations, resistance training may indirectly benefit pulmonary outcomes. By enhancing overall functional capacity and reducing ventilatory demand during daily activities, resistance training alleviates dyspnea and fatigue [118,119]. Moreover, preliminary data suggest that resistance exercise can modulate inflammatory cytokine profiles and improve circulating antioxidant levels, indicating systemic benefits beyond localized muscle strengthening [120,121,122].

Taken together, resistance training provides a targeted approach to addressing COPD-related muscle dysfunction and systemic weakness. When integrated with aerobic exercise, it offers synergistic improvements in physical performance, quality of life, and potentially disease-modifying pathways, making it an indispensable component of comprehensive pulmonary rehabilitation programs.

### 3.3. Comparative Efficacy of HIIT and MICT in COPD Rehabilitation

HIIT and MICT represent two widely investigated aerobic modalities in COPD rehabilitation. While both improve exercise capacity and quality of life, they differ in physiological demands, patient tolerance, and underlying adaptations.

MICT, typically involving prolonged cycling or treadmill walking at 50–70% of maximal workload, is a long-standing and effective method for pulmonary rehabilitation. MICT enhances cardiovascular endurance, reduces ventilatory inefficiency, and increases skeletal muscle oxidative capacity [123]. It is generally well tolerated and safe for patients across different COPD severities, making it a foundational modality in clinical practice. HIIT involves repeated short bursts of exercise at near-maximal intensity, interspersed with recovery periods. Recent studies suggest that HIIT may induce more rapid improvements in exercise tolerance and skeletal muscle metabolism compared to MICT, despite lower total exercise volume [124,125]. HIIT has been shown to reduce dynamic hyperinflation and ventilatory limitation more effectively, thereby allowing COPD patients to achieve higher training intensities with less dyspnea [126,127,128]. Moreover, HIIT enhances mitochondrial biogenesis, increases oxidative enzyme activity, and modulates systemic inflammation more robustly than MICT in certain populations [124,129,130,131]. HIIT has also been associated with greater activation of AMPK-PGC1α signaling, suggesting superior capacity to remodel energy metabolism [125,132,133,134].

Despite its advantages, HIIT may pose challenges for severely debilitated COPD patients due to higher perceived exertion and potential cardiovascular risks. Thus, patient-specific factors including baseline lung function, comorbidities, and training adherence should guide the choice between HIIT and MICT. Increasingly, hybrid approaches that combine interval and continuous elements are being explored to balance efficacy and tolerability.

In summary, both HIIT and MICT improve exercise tolerance and systemic health in COPD, but HIIT may provide more potent metabolic and anti-inflammatory benefits, whereas MICT ensures broad applicability and long-term sustainability. Tailoring exercise prescriptions to individual patient needs remains a central challenge in optimizing COPD rehabilitation outcomes.

## 4. Epigenetic Basis of Exercise Benefits in COPD

While the clinical and physiological benefits of exercise in COPD are well established, the molecular mechanisms that underpin these improvements remain an area of intense investigation. Among the various biological pathways, epigenetic regulation has emerged as a promising explanation for how physical activity exerts long-lasting and systemic protective effects.

In COPD, cigarette smoke, oxidative stress, and chronic inflammation disrupt the normal balance of epigenetic marks, leading to persistent activation of pro-inflammatory genes, impaired antioxidant defenses, and accelerated cellular senescence [135,136]. Exercise is increasingly recognized to counteract these maladaptive patterns by modulating epigenetic programs in lung tissue, skeletal muscle, and circulating immune cells.

Mechanical load, calcium flux, and metabolic stress generated during exercise activate upstream pathways such as AMPK–PGC-1α, CaMKII, and redox-sensitive NRF2 signaling [20,21,137,138]. These cascades converge on nuclear epigenetic regulators, influencing the activity of DNMTs (DNMT1; DNMT3A/3B), TET dioxygenases, and histone deacetylases including HDAC2 and HDAC3 [139,140]. Transcription factors such as PGC-1α, MEF2, NRF2, and NF-κB further integrate these signals by recruiting or displacing epigenetic enzymes at specific loci to fine-tune inflammatory and antioxidant gene expression [141,142]. Importantly, we also note that not all studies report robust epigenetic responses; several short-term or low-volume exercise interventions show minimal or no detectable methylation changes [138,143], underscoring heterogeneity in training protocols and tissue-specific responses.

Different exercise modalities, intensities, and durations produce heterogeneous epigenetic outcomes. Multi-week endurance training at moderate-to-high intensity consistently induces coordinated hypomethylation at genes related to mitochondrial biogenesis and oxidative metabolism in human skeletal muscle [138]. Progressive resistance training for 6–12 weeks elicits distinct and reproducible methylation remodeling at loci governing muscle growth and structural adaptation [143]. Acute bouts of exercise can trigger rapid methylation changes in isolated immune cell subsets or skeletal muscle, although studies analyzing whole PBMCs or global methylation frequently report no detectable modification after a single session, emphasizing the importance of tissue specificity and sampling time [144,145]. In COPD, small pulmonary rehabilitation cohorts show shifts in global or peripheral methylation after multi-week programs, although measurements remain blood-based and lack promoter-resolved lung data [146]. Collectively, available evidence suggests that sustained endurance or resistance training more reliably remodels epigenetic landscapes compared with single acute sessions, but inter-study heterogeneity and methodological limitations preclude firm dose–response prescriptions at present.

**Figure 3:**
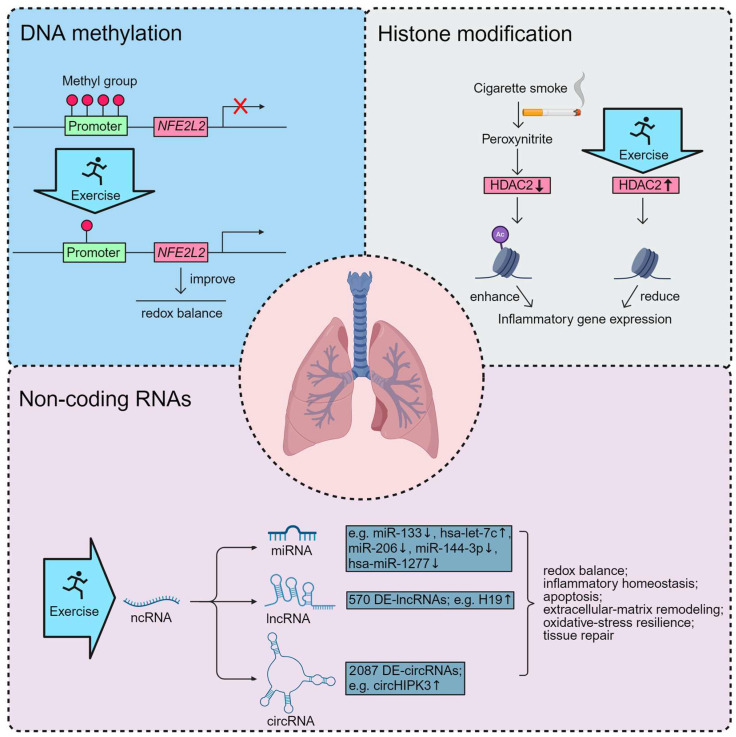
Exercise-induced epigenetic modulation in COPD. Created with BioRender.com, der, b. (2025) https://BioRender.com/my4a89c (accessed on 4 November 2025).

Recent studies suggest that aerobic exercise can restore HDAC2 activity, thereby enhancing anti-inflammatory responses, while resistance training and interval training may modulate DNA methylation profiles of genes related to oxidative stress and mitochondrial biogenesis. In addition, exercise-induced non-coding RNAs have been implicated in regulating pathways of apoptosis, autophagy, and tissue repair. Collectively, these findings highlight that the benefits of exercise extend beyond functional adaptations to include epigenetic remodeling of key molecular networks that underlie COPD pathology (Figure 3).

### 4.1. Exercise-Induced DNA Methylation in COPD

Evidence directly linking exercise to DNA methylation changes in COPD remains limited, but several studies provide preliminary and mechanistic insights. In a pilot study of COPD patients enrolled in an eight-week supervised pulmonary rehabilitation program, da Silva et al. reported an acute decrease in global DNA methylation after the first exercise session and a progressive reduction in baseline circulating pro-inflammatory cytokines (e.g., IL-6 and IL-8) after completion of the program, suggesting that exercise produces measurable changes in peripheral epigenetic markers alongside systemic inflammatory modulation [146]. To more accurately reflect the available evidence, this pilot study represents the only direct human COPD dataset assessing DNA methylation responses to exercise, and it measured global rather than locus-specific methylation. Thus, no published human study has demonstrated promoter-resolved methylation remodeling in COPD lung or airway tissues following exercise.

Independent work has demonstrated that hypermethylation of the *NFE2L2* promoter contributes to reduced Nrf2 expression and impaired antioxidant defenses in COPD lungs, linking locus-specific methylation changes to disease-relevant redox dysfunction [23]. Complementary animal experiments demonstrate that treadmill exercise increases muscle-derived mediators (for example, irisin), upregulates pulmonary Nrf2 and HO-1 expression, and mitigates CS–induced emphysematous changes, thereby providing tissue-level evidence that exercise engages Nrf2-centered antioxidant pathways relevant to COPD protection [147]. These preclinical findings support the biological plausibility that exercise could influence methylation states of antioxidant genes, although direct evidence for demethylation of *NFE2L2* or other loci in COPD lung tissue following exercise is currently lacking.

Mechanistically, exercise has been shown in non-pulmonary models to reverse promoter hypermethylation of *NFE2L2* and to reduce aberrant DNA methyltransferase (DNMT) expression, leading to restored Nrf2 transcription and improved redox balance [148]. In addition, several intervention studies report locus-specific methylation or expression changes that connect epigenetic modulation to clinically relevant targets. For example, exercise-associated demethylation and transcriptional recovery of antioxidant genes (*NFE2L2*, *SOD2*) and reduced promoter methylation at genes involved in mitochondrial function have been documented in training studies in non-pulmonary tissues and are congruent with the upregulation of Nrf2-dependent transcripts observed in smoke-exposed animals following exercise [148,149]. However, because these observations originate from non-pulmonary or non-COPD models, they should be considered supportive rather than indicative of a confirmed mechanism in COPD. While these findings jointly suggest that exercise can influence redox- and inflammation-related pathways that are also epigenetically regulated in COPD, they do not establish a causal sequence linking exercise to locus-specific DNA methylation remodeling in COPD lung tissue. Therefore, the current evidence should be viewed as hypothesis-generating, and definitive demonstration of methylation-mediated protection will require studies that pair well-defined exercise interventions with tissue-resolved epigenomic profiling in human COPD.

### 4.2. Exercise-Induced Histone Modification in COPD

Current evidence directly demonstrating that exercise ameliorates COPD through regulated histone modifications is limited. Clinical pulmonary rehabilitation trials in COPD have documented reductions in systemic inflammatory mediators and alterations in peripheral epigenetic markers, but locus-specific histone mark mapping at pro-inflammatory or antioxidant gene promoters has not been reported in these interventions [146]. To our knowledge, no study has evaluated histone acetylation or methylation patterns in lung or airway tissues before and after exercise in COPD patients, representing a major evidence gap.

Observational studies have consistently documented reduced histone deacetylase activity in COPD, notably decreased HDAC2 expression and lowered class III deacetylase SIRT1 levels in lung and peripheral tissues, changes that are mechanistically linked to heightened inflammatory signaling, oxidative stress and corticosteroid resistance in patients [42,150,151,152]. Exercise and physical activity are known to influence histone marks in non-pulmonary tissues. Human and animal studies have reported exercise-associated increases in activating histone acetylation (e.g., H3K9ac, H3 acetylation) and enrichment of activating methylation marks (e.g., H3K4me3) at genes governing mitochondrial biogenesis and metabolic adaptation [153,154,155]. While these findings underscore the plausibility that exercise may also influence chromatin states in COPD, the absence of direct histone mapping in lung tissues precludes mechanistic conclusions. Importantly, although HDAC2 and SIRT1 reductions are central to COPD pathobiology, no available study has demonstrated that exercise restores the expression or activity of these enzymes in COPD lung tissue, and therefore they should be regarded as potential mechanistic targets rather than established mediators of exercise benefit.

In CS–exposed rodent models, exercise interventions reproducibly activate cytoprotective pathways such as Nrf2/HO-1 and ameliorate emphysematous pathology, providing tissue-level evidence of benefit. However, these reports have generally not included ChIP-based or other locus-specific assays of histone marks at pro-inflammatory or antioxidant gene promoters, so the chromatin-level mediators remain uncharacterized [147]. Mechanistic animal work linking histone-modifying enzymes to smoke-induced muscle wasting and inflammation further underscores candidate mediators (for example, HDAC2 involvement in skeletal muscle dysfunction), suggesting targets for focused chromatin analyses in intervention studies [100]. Taken together, the literature supports the hypothesis that exercise can reprogram chromatin states in ways that favor antioxidant and anti-inflammatory gene expression, but definitive demonstration in COPD requires intervention studies that pair well-defined exercise prescriptions with tissue-resolved, locus-specific histone profiling and matched functional endpoints.

### 4.3. Exercise-Induced ncRNAs in COPD

Emerging evidence indicates that exercise modulates the expression of ncRNAs, including miRNAs, lncRNAs, and circRNAs, which may contribute to protective effects in COPD. Recent human data provide preliminary but direct evidence that exercise modulates specific miRNAs associated with COPD pathophysiology. In an observational cohort, miR-133 and miR-206, two muscle-enriched myomiRs involved in skeletal-muscle differentiation and regeneration, were negatively correlated with daily physical activity levels in patients with COPD, suggesting a link between reduced activity and muscle dysfunction–related miRNA expression [53]. In a pilot interventional study, 12 weeks of individualized aerobic training in patients with moderate-to-severe COPD led to downregulation of miR-144-3p and hsa-miR-1277 and upregulation of hsa-let-7c in peripheral blood [156]. These alterations coincided with improvements in exercise tolerance and reductions in circulating IL-6 and TNF-α. Notably, the upregulation of let-7c, a miRNA known to target inflammatory mediators [157], and the suppression of miR-144-3p, previously linked to oxidative stress and erythroid dysfunction in COPD [158], imply that aerobic training exerts systemic anti-inflammatory and cytoprotective effects through miRNA regulation. Although the sample sizes were small and locus-specific mechanistic validation is lacking, these studies provide the currently available evidence that structured exercise interventions can reprogram disease-relevant miRNA networks in COPD patients, potentially contributing to improved redox and inflammatory homeostasis.

Evidence from animal models corroborates these findings and extends them to lncRNAs and circRNAs. Recent animal studies provide direct evidence that exercise modulates lncRNA expression in smoke-exposed COPD models and that these changes may contribute to tissue protection. For example, in a mouse model of CS-induced COPD, aerobic training was shown to reduce lung expression of lncRNA H19, a lncRNA previously found to promote ROS generation, epithelial–mesenchymal transition (EMT) and lung injury, and this reduction correlated with decreased inflammation, suppressed EMT marker α-SMA, and preservation of alveolar architecture [95]. Moreover, a comprehensive transcriptomic profiling study of COPD patients after 12 weeks of aerobic training revealed differential expression of 570 lncRNAs in peripheral blood, concurrent with changes in mRNA and circRNA networks, implying that exercise broadly alters lncRNA landscapes in COPD contexts [156]. In parallel, 2087 circRNAs were found to be differentially expressed in peripheral blood post-training in this study, supporting the idea that exercise affects circRNA networks in COPD [156]. Functional studies in related injury models indicate that circRNAs (for example circHIPK3) can sponge pro-inflammatory miRNAs (such as miR-21), thereby reducing NF-κB activation and oxidative stress in target cells [95,159]. However, these functional observations derive from related injury models rather than COPD itself, and thus cannot be taken as evidence that similar ncRNA-dependent mechanisms mediate exercise-induced protection in COPD lung tissue.

Collectively, these studies provide direct evidence that exercise remodels ncRNA networks in COPD, influencing pathways involved in inflammation, oxidative stress, apoptosis, and tissue repair. While the data remain limited, these findings support the concept that ncRNA-mediated regulation represents a potential mechanism by which exercise confers protective effects in COPD. Future studies employing longitudinal human cohorts and integrated multi-omics approaches will be critical to delineate the precise ncRNA networks modulated by exercise and their functional relevance to clinical outcomes.

## 5. Discussion

This review synthesizes emerging evidence that structured physical activity influences molecular processes relevant to COPD through multiple epigenetic layers. Clinical pulmonary rehabilitation and supervised exercise consistently reduce systemic inflammatory mediators and improve functional outcomes in COPD; in several small human studies these clinical benefits have been accompanied by changes in peripheral epigenetic markers, most notably at the level of global DNA methylation and circulating ncRNAs [146,156]. Complementary preclinical work demonstrates that exercise upregulates cytoprotective signaling such as the Nrf2/HO-1 axis and attenuates CS–induced emphysematous changes in rodent models, establishing tissue-level molecular and morphological correlates of exercise benefit [147]. Independent molecular studies have documented disease-associated epigenetic substrates in COPD lungs and airways, including promoter hypermethylation of antioxidant regulators and perturbations in histone-modifying enzymes such as HDAC2 and SIRT1 [23,42,95,150]. Together, these observations identify epigenetic substrates that could represent mechanistic points of interaction for exercise, although direct evidence linking exercise to targeted remodeling of these marks in COPD lungs remains unavailable.

Despite these convergent lines of evidence, the mechanistic picture remains incomplete. No published study to date has established the full causal chain—exercise intervention, locus-specific epigenetic remodeling in the lung, and consequent, reproducible improvement in COPD clinical end points—in a single, well-powered human or animal experiment. Most human trials that incorporate epigenetic measurements have been small and focused on peripheral samples [146,156]. Even when epigenetic changes are observed in PBMCs or plasma, whether these alterations parallel lung-resident epigenetic remodeling remains unknown, as concordance between blood and lung tissues has not been systematically evaluated in COPD. Moreover, because epigenetic marks are inherently dynamic, most existing studies lack multi-time-point sampling, preventing differentiation between transient exercise-induced fluctuations and durable adaptations that may mediate clinical improvement. Animal studies more readily combine intervention and tissue analysis, but most report pathway activation and phenotypic rescue without concurrent, genome-wide or targeted histone/chromatin profiling, limiting the ability to determine whether specific histone modifications mediate exercise responses [147]. Studies of ncRNAs are promising and include both human and murine data showing exercise-associated modulation of miRNAs, lncRNAs and circRNAs. However, these datasets lack longitudinal resolution and functional perturbation (gain- or loss-of-function) evidence that could establish causal mediation [95,156,160].

In addition to epigenetic mechanisms, COPD pathogenesis is strongly shaped by protease–antiprotease imbalance, chronic oxidative stress, and persistent inflammation. Elevated levels of matrix metalloproteinase-9 (MMP-9) and an increased MMP-9/TIMP-1 ratio are consistently associated with emphysema severity and extracellular matrix degradation in COPD [161,162]. Likewise, impaired Nrf2-dependent antioxidant responses contribute to heightened oxidant burden and tissue injury [163]. Exercise interventions, although not directly shown to modulate protease activity at the epigenetic level, have been reported to attenuate systemic inflammation and improve redox homeostasis in COPD, partly through upregulation of cytoprotective pathways such as Nrf2/HO-1 in preclinical smoke-exposed models [147]. Furthermore, evidence from non-COPD contexts indicates that resistance training can modulate MMP-2 and MMP-9 expression in skeletal muscle, visceral adipose tissue and circulation, supporting a plausible link between improved protease–antiprotease balance and regular physical activity, though studies directly evaluating these pathways in COPD remain lacking [164]. Integrating these findings, exercise may exert multi-layered regulatory effects including reduce inflammatory signaling, enhance antioxidant capacity, and potentially influence protease-related pathways, thereby creating a biological context in which epigenetic remodeling could contribute to durable improvements in pulmonary homeostasis. However, definitive mechanistic links among exercise, epigenetic remodeling, and protease/oxidant homeostasis in COPD remain to be demonstrated.

These limitations point to specific priorities for future research. Clinical trials should prospectively embed standardized epigenetic end points, collect appropriately matched biospecimens (for example, PBMCs, bronchial brushings or BAL cells, and skeletal muscle biopsies) at harmonized timepoints (baseline, early response, end of intervention and follow-up), and pair molecular profiling with rigorous clinical outcomes (spirometry, symptom scores, exercise capacity, and quantitative imaging). Multi-omics approaches that combine whole-genome bisulfite sequencing or array-based methylome profiling, ChIP-seq for key histone marks and chromatin modifiers, and small/total RNA sequencing for ncRNAs will be essential to map coordinated regulatory networks. In parallel, mechanistic animal studies should integrate locus-specific epigenetic assays with genetic or epigenetic perturbation (e.g., DNMT/HDAC/SIRT modulation, CRISPR-Cas9–based epigenome editing) to test whether reversing a given epigenetic lesion is necessary and sufficient for exercise-mediated phenotypic rescue. Careful attention to exercise prescription (modality, intensity, duration), participant phenotyping (inflammatory status, comorbidity, medication use), and statistical power will be critical to resolve heterogeneity and to identify subgroups most likely to derive epigenetic and clinical benefit.

From a translational perspective, the current literature supports cautious optimism rather than immediate clinical application. Epigenetic signatures, if validated in larger, longitudinal cohorts, could become useful biomarkers for stratifying response to pulmonary rehabilitation or for monitoring biological responses to exercise. Likewise, combinatorial strategies that pair rehabilitation with pharmacologic modulators of epigenetic enzymes remain an intriguing hypothesis, but such approaches should be pursued only after mechanistic proof of concept is obtained. In the near term, investigators running pulmonary rehabilitation trials should consider routine collection of biospecimens and standardized epigenetic assays to build the evidence base required for future precision rehabilitation.

In summary, exercise-mediated modulation of epigenetic mechanisms represents a biologically plausible route by which physical activity can influence inflammation, redox balance and tissue integrity in COPD. Existing clinical and preclinical studies provide initial support for this model but do not yet establish locus-specific causal mediation. Addressing this gap will require adequately powered, tissue-resolved, and mechanistically informed studies that integrate multi-omics profiling with robust phenotyping and interventional perturbations.

## 6. Conclusions

Exercise provides multifaceted benefits in COPD, extending beyond improvements in physical performance to modulation of key molecular pathways involved in disease progression. Emerging evidence demonstrates that these protective effects are mediated, at least in part, through epigenetic mechanisms, including DNA methylation, histone modifications, and ncRNA regulation. Exercise may restore aberrant methylation patterns, rebalance histone acetylation and methylation at critical gene loci, and fine-tune ncRNA networks, collectively mitigating inflammation, oxidative stress, and tissue remodeling. The integration of clinical and preclinical findings underscores the potential of exercise as a disease-modifying intervention, offering both functional and molecular benefits. These insights highlight the importance of incorporating structured physical activity into COPD management and provide a foundation for personalized rehabilitation strategies informed by epigenetic profiling.

## 7. Future Directions

Future research should focus on elucidating the causal relationships between exercise-induced epigenetic modifications and clinical outcomes in COPD. Longitudinal studies with larger patient cohorts and multi-tissue analyses are needed to capture the systemic impact of exercise and to identify biomarkers predictive of individual responsiveness. Multi-omics approaches, integrating methylomics, histone modification mapping, transcriptomics, and ncRNA profiling, will be critical for constructing a comprehensive view of exercise-mediated epigenetic regulation. Additionally, comparative studies exploring different exercise modalities, intensities, and durations will help optimize rehabilitation protocols. Investigating combinatorial strategies that integrate pharmacological epigenetic modulators with exercise interventions may further enhance therapeutic efficacy. Ultimately, a deeper understanding of epigenetic mechanisms will facilitate precision rehabilitation, enabling tailored exercise prescriptions that maximize molecular and functional benefits in COPD patients.

## Data Availability

No new data were created or analyzed in this study.

## Abbreviations

The following abbreviations are used in this manuscript:

circRNAs	Circular RNAs
COPD	Chronic Obstructive Pulmonary Disease
CS	Cigarette Smoke
DNMT	DNA Methyltransferase
EMT	Epithelial–Mesenchymal Transition
EWAS	Epigenome-Wide Association Studies
HDACs	Histone Deacetylases
HIIT	High-Intensity Interval Training
lncRNAs	Long Non-Coding RNAs
MICT	Moderate-Intensity Continuous Training
miRNAs	MicroRNAs
ncRNAs	Non-Coding RNAs
